# Supplementary material for: Memory persistence and differentiation into antibody-secreting cells accompanied by positive selection in longitudinal BCR repertoires
Source: eLife. 2022 Sep 15;11:e79254. doi: 10.7554/eLife.79254 (PMC9525062; doi:10.7554/eLife.79254)
Supplement: Figure 5—source data 1. [file elife-79254-fig5-data1.docx]

**Table S1.** Examples of divergent (*D*) and polymorphic (*P*) sites as calculated for the McDonald-Kreitman (MK) test. Synonymous and nonsynonymous substitutions from germ-line sequence are underlined and boldfaced, correspondingly.

| **Codon #** | **i** | | | **j** | | | **q** | | | **r** | | |
| --- | --- | --- | --- | --- | --- | --- | --- | --- | --- | --- | --- | --- |
| **Germline** | c | g | c | g | - | - | c | t | a | a | a | t |
| **MRCA** | c | g | t | g | t | a | c | t | c | a | **g** | t |
| **Clonotypes in the clonal lineage** | c  c  c  c | g  **t**  g  **a** | t  t  t  t | g g  g  g | t  t  t  t | a  a  a  a | c  c  c  c | t  t  t  t | c  a  c  c | a  a  a  a | **g** **g**  **c**  **c** | t  t  t  t |
| ***Nonsynonymous divergence (Dn)*** | 0 | 0 | 0 | - | - | - | 0 | 0 | 0 | 0 | **1** | 0 |
| ***Synonymous divergence (Ds)*** | 0 | 0 | 1 | - | - | - | 0 | 0 | 0 | 0 | 0 | 0 |
| ***Nonsynonymous polymorphism (Pn)*** | 0 | **2** | 0 | - | - | - | 0 | 0 | 0 | 0 | **1** | 0 |
| ***Synonymous polymorphism (Ps)*** | 0 | 0 | 0 | - | - | - | 0 | 0 | 1 | 0 | 0 | 0 |
| **Comment** | example of codon represented by multiple variants in the clonal lineage (*i.e.* with the multiallelic site) | | | example of codon excluded from analysis because of unknown germline sequence for the site | | | example of codon where divergence is not counted because of presence of the germline variant among sequence variants in the lineage | | | example of codon where divergence is counted because there are no clonotypes identical to the germline sequence | | |
