# Supplementary material for: Memory persistence and differentiation into antibody-secreting cells accompanied by positive selection in longitudinal BCR repertoires
Source: eLife. 2022 Sep 15;11:e79254. doi: 10.7554/eLife.79254 (PMC9525062; doi:10.7554/eLife.79254)
Supplement: Figure 5—source data 2. [file elife-79254-fig5-data2.docx]

**Table S2.** MK test results under different inclusion criterion for clonal lineages from HBmem and LBmem clusters, which makes it possible to deal with zero values in G-MRCA nonsynonymous or synonymous divergence. The LBmem cluster demonstrated consistent results of the MK test under all inclusion criteria, and the α of joined inside cluster divergence (combined SHM for all lineages belonging to the cluster) corresponds well to the median α among clonal lineages. The HBmem cluster is better suited for this type of filter, since it generally has much lower G-MRCA distance, and some clonal lineages have no divergence in MRCA from reconstructed portions of the germline sequence. Estimated α on joined cluster divergence in the HBmem cluster varies depending on the type of the filter employed, but is always lower than the α of LBmem. Additionally, consideration of all clonal lineages with addition of pseudocounts to *Dn* and *Ds* produces a negative median α, because the α of a clonal lineage with zero G-MRCA distance will always produce a negative α.

| **Inclusion criteria** | All clonal lineages. Pseudocounts are added to *Dn* and *Ds* to deal with zero values in the MK test of distinct clonal lineages. | | Clonal lineages with nonzero G-MRCA distance (at least one nonsynonymous or synonymous substitution). Pseudocounts are added to *Dn* and *Ds* to deal with zero values in the MK test of distinct clonal lineages. | | Clonal lineages with at least one nonsynonymous and synonymous substitution. No pseudocounts in *Dn* and *Ds* are required. | |
| --- | --- | --- | --- | --- | --- | --- |
| **Cluster** | HBmem | LBmem | HBmem | LBmem | HBmem | LBmem |
| **# of filtered clonal lineages** | 138 | 52 | 68 | 49 | 18 | 29 |
| **Median α** | -0.46 | 0.55 | 0.18 | 0.57 | - 0.07 | 0.54 |
| **Mann-Whitney test** | p = 2.9ᐧ10^-11^ | | p = 4.8ᐧ10^-6^ | | p = 0.0028 | |
| **MK test on joined diversity of the cluster** | α = 0.58 p = 4.97ᐧ10^-7^ | α = 0.65 p < 2.2ᐧ10^-16^ | α = 0.61 p = 6.05ᐧ10^-8^ | α = 0.66 p < 2.2ᐧ10^-16^ | α = 0.26 p = 0.1004 | α = 0.56 p = 2.05ᐧ10^-10^ |
